# The detrimental effect of donor-specific antibodies is irrespective of its level in highly-immunized living donor kidney transplant recipients: A case-control series

**DOI:** 10.3389/fimmu.2022.1093359

**Published:** 2023-01-10

**Authors:** T. Tramper, D. L. Roelen, S. H. Brand-Schaaf, J. A. Kal-van Gestel, M. M. L. Kho, M. E. J. Reinders, J. I. Roodnat, J. van de Wetering, M. G. H. Betjes, A. E. de Weerd

**Affiliations:** ^1^ Erasmus Medical Center Transplant Institute, Department of Internal Medicine, University Medical Center Rotterdam, Rotterdam, Netherlands; ^2^ HLA Laboratory, Department of Immunology, Leiden University Medical Center, Leiden, Netherlands

**Keywords:** kidney transplantation, donor-specific antibodies, antibody-mediated (ABMR), panel-reactive antibodies, graft survival

## Abstract

**Background:**

The impact of donor-specific antibodies (DSA) in (highly-) immunized living donor kidney transplant recipients is reported differentially in various patient cohorts.

**Methods:**

We have performed a retrospective analysis of all consecutive HLA-incompatible living donor kidney transplant recipients in our center between 2010-2019. Recipients who underwent plasmafiltration for a positive CDC-crossmatch were excluded. For each DSA+ recipient (DSA+), one immunized recipient without DSA (pPRA+) and two non-immunized recipients (pPRA-) were included. Patient and graft survival were analyzed and a subgroup analysis of DSA+ recipients was performed.

**Results:**

For 63 DSA+ recipients, 63 PRA+ and 126 PRA- recipients were included. 26 (41%) had class I, 24 (38%) class II and 13 (21%) combined HLA class I and II DSA. Death-censored graft survival was inferior in DSA+ recipients compared to pPRA+ (HR 2.38 [95% CI 1.00-5.70]) as well as to pPRA- (HR 3.91 [1.86-8.22]). In multivariate analysis, DSA remained of negative influence on death-censored graft survival. Flowcytometric crossmatch, MFI value, HLA class and origin of DSA were not of significant impact.

**Conclusion:**

In our cohort of (highly-) immunized recipients, pretransplant DSA led to inferior death-censored graft survival. There were no “safe” DSA characteristics since only DSA *per se* impacted death-censored graft survival.

## Introduction

Kidney transplantation is the preferred treatment for end-stage renal disease, but shortage of available kidney donors and a limited survival of kidney allografts increase the demand of an already limited donor pool (1–5). Donor-specific antibodies (DSA) against HLA may arise after a previous transplantation, pregnancy or blood transfusion. As the presence of DSA pretransplantation can lead to antibody-mediated rejection (ABMR) and subsequently graft-loss, it is generally avoided in transplantation.

The pre-transplantation workup consists of different assays to detect DSA including a cytotoxicity-dependent (CDC) crossmatch, flowcytometric (flow) B- and T-cell crossmatches and the Luminex single antigen bead (SAB) assay. There is an increasing sensitivity to detect anti-HLA antibodies in these assays (6), corresponding to different risk profiles for ABMR post-transplantation.

Although DSA are considered harmful, the impact of DSA on rejection and graft survival is differentially reported (7–11). The Dutch PROCARE consortium reported a negative effect of DSA in deceased donor transplantation only, not in living donor transplantation (7, 8). Higgins et al. did not find a negative impact of DSA on graft outcomes provided that the CDC-crossmatch was negative (12). In our center, where the majority of kidney transplants are from living donors, pretransplant DSA have not been considered an absolute contra-indication in the absence of a compatible living donor and when there is a negative CDC-crossmatch. In addition, (highly-) immunized recipients and frequent repeat transplantations are common in our living donor program. Since outcomes in literature vary, we hypothesized that the impact of pretransplant DSA in living donor kidney transplantation differs according to immunization level. The aim of this study is to determine the effect of DSA on rejection, death-censored graft survival (DCGS) and patient survival in our (highly-) immunized cohort of living donor kidney transplant recipients. Therefore, we included DSA-positive transplantations and used both immunized and non-immunized recipients as control groups. Furthermore, we aim to identify factors that might explain the varying effect of DSA on allograft outcomes, such as level and origin of DSA.

## Patients and methods

### Recipients

We analyzed all consecutive living donor kidney transplant recipients with pre-transplant DSA in the Erasmus Medical Center between 1-1-2010 and 31-12-2019. Adult recipients of 18 years and older were included. ABO-incompatible kidney transplant recipients were also included in our analysis. Recipients who had a positive CDC-crossmatch, for which they were treated with desensitization by plasmafiltration, were excluded. We performed a retrospective case-control study by matching each DSA+ recipient to one immunized recipient with comparable level of peak panel-reactive antibodies (pPRA+) and to two non-immunized kidney transplant recipients, with PRA <6% (pPRA-), with a ratio of 1:1:2 respectively. Matching criteria were year of transplantation (+1 or -1 year), pPRA% (for highly-immunized controls), age of donor and recipient and the relationship between donor and recipient (child or spouse to mother, other family, non-related). Written informed consent was obtained from all recipients.

This trial was approved by the institutional review board of the Erasmus Medical Center (MEC-2021-0357). The clinical and research activities being reported are consistent with the Principles of the Declaration of Istanbul as outlined in the ‘Declaration of Istanbul on Organ Trafficking and Transplant Tourism’ and the Declaration of Helsinki as outlined in the ‘Ethical Principles for Medical Research Involving Human Subjects’.

### Treatment

Standard induction therapy consisted of basiliximab in all three groups. Formerly, the clinical protocol did not provide for a differential approach in DSA+ transplantation, and administration of depleting induction therapy, such as alemtuzumab or recombinant anti-thymocyte globulin (rATG), was left at the discretion of the treating physician. Maintenance immunosuppression was similar in the groups and consisted of tacrolimus in combination with mycophenolic acid and prednisolone. In case of an ABO-incompatible donor, desensitization consisted of immunoadsorption combined with rituximab until 2015 and alemtuzumab thereafter (13). CDC-crossmatch positive transplantations were desensitized by plasmafiltration and excluded in the current analysis.

Allograft biopsies were performed in case of deteriorating kidney function or proteinuria. No surveillance biopsies were performed in this cohort. Rejection was diagnosed using Banff revised ‘17 histology criteria (14). In case of ABMR, treatment consisted of pulse methylprednisolone and high-dose IVIG with or without plasmapheresis and lymphocyte depleting therapy. Cases of T-cell mediated rejection (TCMR) were treated with high dose pulse methylprednisolone. Banff IIA and Banff IIB vascular TCMR were treated with methylprednisolone and in case of refractory vascular rejection with methylprednisolone in combination with either rATG or alemtuzumab (15).

### Identification of DSA

A, B, C, DRB1, DRB3, DRB4, DRB5, DQA, DQB, DPA and DPB were considered for DSA testing. During the study period, the methods to detect HLA antibodies have changed from ELISA screening, to Luminex screening and subsequently Luminex Single Antigen Bead (SAB) testing. LifeCodesIn case of a positive screening this was followed by antibody identification by SAB assay of either Lifecodes or OneLambda. For the Lifecodes SAB test, data were analyzed using MATCHIT! Antibody software version 1.3.1 (Immucor) and results were shown as mean fluorescence intensity (MFI) values, background corrected. Cut-offs were bead-specific in combination with a raw MFI of more than 750. For OneLambda, data were analyzed using HLA FUSION antibody software version 3.4.18 (One Lambda). Results were shown as normalized MFI. For the SAB assay, in 56% of recipients a OneLambda kit was used and in 44% an LifeCodes kit. Furthermore, in case of a positive SAB result, a T-lymphocyte as well as a B-lymphocyte flow-crossmatch was performed. For each patient, a CDC-crossmatch was performed, with and without DTT, in order to analyze only IgG antibodies. The origin of DSA was divided in four categories; DSA against a repeated HLA-mismatch (RMM), based on HLA type of prior donors as listed in the Eurotransplant registry; DSA originating from pregnancy (female recipients with a pregnancy, where the HLA-typing of child or partner matched the DSA); DSA in males with no RMM; and DSA in females with no RMM and no HLA typing of partner or child matching the DSA.

### Definition of immunization

The definition of immunization (PRA+) was defined as peak PRA ≥6% resulting from current and historical HLA antibody screening. DSA+ was defined as the presence of DSA in pre-transplant samples of which the results were already known pre-transplant. For the purpose of this study, in all PRA+ patients, the pre-transplant samples were retested with Luminex SAB to rule out DSA despite negative Luminex screening.

### Outcomes

The primary outcome is DCGS. Secondary outcomes are patient survival and biopsy-proven acute rejections (BPAR) according to Banff classification (14). In this study ABMR also included ABMR histology in the absence of DSA (16).

### Data collection

Data were retrieved from both the Dutch Transplant Registry (“Nederlandse Orgaan Transplantatie Registratie, NOTR)” and the hospital electronic patient files.

### Statistical analysis

For descriptive statistics categorical data were compared with z-tests for proportions. Median values were used when data did not follow a normal distribution, determined with the Shapiro-Wilk test. Comparison of numerical data between groups was performed with an independent samples t-test for two groups and one way ANOVA for three groups in case of equal variances. In the case of non-equal variances comparisons were made with a Mann-Whitney-U test for two groups and a Kruskal-Wallis test for three groups. Equal variances were investigated by use of the Levene’s test. Kaplan-Meier analysis was performed for survival and rejection data. A Cox proportional hazard model was used to compare survival and rejection differences. Univariate analyses were performed using the following pre-defined variables: pPRA, HLA-mismatches on A/B/DR, year of transplantation, recipient-donor relation (child or spouse to mother, other family, non-related), pre-emptive transplantation, number of transplantations (one, two or more), dialysis vintage, age, sex and blood group of donor and recipient, ABO-compatibility, primary kidney disease, body mass index (BMI), induction therapy and (initial) immunosuppression. For main outcomes, associations with a p-value of p <0.1 were included in multivariate analysis, as well as age and sex of recipient. The lowest value of either the square root of the number of events or one tenth of the number of events was used as the maximal amount of variables in multivariate analysis. Pearson’s correlation and stratified Kaplan-Meier analyses were performed in order to examine interactions between variables. Collinearity between variables was excluded. In the case of a correlation with r >0.5 and presumption of a synergistic effect, an interaction term was used in the multivariate analysis with backwards conditional method. A predefined subgroup analysis of all DSA+ patients was performed to decipher the impact of DSA characteristics on outcomes after HLA-incompatible living donor kidney transplantation. A sensitivity analysis was performed either excluding recipients whose DSA were tested with OneLambda or excluding those whose DSA were tested with Lifecodes. Analyses were performed with SPSS statistics version 28.0.1.0 (142).

## Results

### Comparison of DSA positive recipients with matched immunized (pPRA+) and non-immunized (pPRA-) controls

#### Baseline characteristics

Between 2010 and 2019, 75 living donor DSA+ kidney transplantations were performed in our center. Twelve were CDC+ combinations for which desensitization was performed and these were excluded. The 63 included recipients were matched to 63 evenly immunized (pPRA+) and 126 non-immunized (pPRA-) recipients (DSA+ n=63; pPRA+ n=63; pPRA- n=126). Median recipient age was 51, 53 and 53 years respectively (p=0.48 and p=0.31, **Table 1**). Median donor age was 50, 48 and 52 years respectively (p=0.69 and p=0.48). Immunized recipients were more often female (respectively 64%, 57% and 22%, p<0.001 for the two immunized groups, DSA+ and pPRA+ versus pPRA-). ABO-incompatibility did not differ significantly between the three groups (5 vs 11 vs 9%). 32% of DSA+ were highly immunized (pPRA>85%) versus 24% of pPRA+ (p=0.32). Mismatches on HLA-A did not differ between the groups, however mismatches on HLA-B and HLA-DR were more common in DSA+ (respectively p=0.02 and p=0.002 vs. pPRA+). Retransplantation was more common in DSA+, with 38 (60%) versus 26 (41%) in pPRA+ (p=0.03). A recipient donor relationship where pregnancy might have induced DSA formation was more frequent in DSA+ (p=0.03 vs. pPRA+ and p=0.001 vs. pPRA-). There were more child to mother and more partner to female combinations in the DSA+ population. In pPRA+, transplantations were more often non-related than in DSA+ and pPRA-. Depleting induction therapy, alemtuzumab or rATG, was administered in a small portion of recipients, and more often in immunized recipients: 12% vs 9% vs 3% in DSA+, PRA+ and PRA- respectively (p=0.01 for immunized versus non-immunized).

**Table 1:** Baseline characteristics of donor-specific antibody (DSA) positive living donor kidney transplant recipients (DSA+) and matched immunized (PRA+) and non-immunized (PRA-) control recipients.

	Immunized		Non-immunized		
	DSA+	PRA+	PRA-	*p-value* DSA+vs. PRA+	*p-value* DSA+ vs. PRA-
**N**	63	63	126		
**Age recipient [median years, IQR]**	51 [34-61]	53 [39-59]	53 (38-62]	0.48	0.31
**Sex recipient [females, %]**	40 [64%]	36 [57%]	40 [22%]	0.47	<0.001
**BMI recipient** **[median, IQR]**	24.5 [22.3-27.7]	26.0 [22.4-30.5]	26.3 [23.4-29.4]	0.35	0.01
**Preemptive transplantation [n, %]**	19 [30%]	18 [29%]	50 [40%]	0.85	0.2
**Time on dialysis (days)** **[median, IQR]**	309 [0-708]	209 [0-470]	178 [0-533]	0.35	0.27
Transplantations [%]					
1^st^	25 [40%]	37 [59%]	116 [92%]	0.03	<0.001
2^nd^	25 [40%]	19 [30%]	9 [7%]	0.26	<0.001
3^rd^	10 [16%]	6 [10%]	1 [1%]	0.29	<0.001
4^th^ or more	3 [5%]	1 [2%]	0 [0%]	0.31	–
Recipient blood group [%]					
A	28 [44%]	27 [43%]	55 [44%]	0.86	0.92
B	10 [16%]	9 [14%]	16 [13%]	0.8	0.55
AB	2 [3%]	3 [5%]	8 [6%]	0.65	0.36
0	23 [37%]	24 [38%]	47 [37%]	0.85	0.92
**Blood group incompatible transplantation [%]**	3 [5%]	7 [11%]	11 [9%]	0.19	0.33
Primary kidney disease [%]					
Diabetic Nephropathy	6 [10%]	4 [6%]	21 [17%]	0.51	0.19
Glomerulonephritis	18 [29%]	20 [32%]	29 [23%]	0.7	0.41
Urological	8 [13%]	5 [8%]	5 [4%]	0.38	0.03
Cystic kidney disease	7 [11%]	6 [10%]	15 [12%]	0.77	0.87
Vascular	13 [21%]	14 [22%]	30 [24%]	0.82	0.62
Hereditary	0 [0%]	1 [2%]	4 [3%]	–	–
Other/Unknown	11 [18%]	13 [21%]	22 [18%]	0.65	1
**Age donor (years) [median, IQR]**	50 [37-59]	48 [41-57]	52 [40-59]	0.69	0.48
**Sex donor [females, %]**	37 [59%]	42 [67%]	73 [58%]	0.36	0.92
Recipient-Donor Relation [%]					
Possible anti-HLA by pregnancy	14 [22%]	5 [8%]	7 [6%]	0.03	0.001
- Child to Mother	7 [11%]	4 [6%]	2 [2%]	0.34	0.004
- Partner to female	7 [11%]	1 [2%]	5 [4%]	0.03	0.06
Family in Law	4 [6%]	1 [2%]	10 [8%]	0.17	0.69
Other Related	17 [27%]	15 [24%]	44 [35%]	0.68	0.27
Other Non-related	28 [44%]	42 [67%]	65 [52%]	0.01	0.35
Mismatches A [%]					
0	13 [21%]	18 [29%]	24 [20%]	0.3	0.82
1	29 [47%]	26 [42%]	71 [58%]	0.59	0.16
2	20 [32%]	18 [29%]	28 [23%]	0.7	0.16
Mismatches B [%]					
0	5 [8%]	15 [24%]	18 [15%]	0.02	0.21
1	27 [44%]	28 [45%]	54 [44%]	0.86	0.96
2	30 [48%]	19 [31%]	51 [42%]	0.04	0.37
Mismatches DR [%]					
0	8 [13%]	23 [37%]	23 [19%]	0.002	0.32
1	29 [47%]	25 [40%]	64 [52%]	0.47	0.5
2	25 [40%]	14 [23%]	36 [29%]	0.03	0.13
Peak PRA [%]					
**Median [IQR]**	75 [35-90]	73 [40-84]	4 [0-4]	1	<0.001
1-5%	4 [6%]	5 [8%]	126 [100%]	0.71	–
6-84%	39 [62%]	43 [68%]	0[0%]	0.49	–
85-100%	20 [32%]	15 [24%]	0 [0%]	0.35	–
**Transplantation Year** **[median, IQR]**	2013 (2011-2014)	2015 (2012-2017)	2013 (2012-2015)	0.03	0.41
Induction therapy [%]					
No induction	1 [2%]	6 [10%]	9 [7%]	0.05	0.11
Basiliximab	53 [84%]	47 [75%]	103 [82%]	0.19	0.69
Rituximab	1 [2%]	4 [6%]	10 [8%]	0.17	0.11
ATG	4 [6%]	2 [3%]	3 [2%]	0.4	0.17
Alemtuzumab	4 [6%]	4 [6%]	1 [1%]	1	0.03
Baseline Immunosuppression [%]					
Corticosteroids, Tacrolimus & Mycophenolic acid	59 [94%]	59 [94%]	120 [95%]	0.65	0.65
Other	4 [6%]	4 [6%]	6 [5%]	0.65	0.65

IQR, inter-quartile range; DSA, donor specific antibodies; PRA, panel reactive antibodies; ATG, anti-thymocyte globulin.

#### Death-censored graft survival

**Figure 1:**
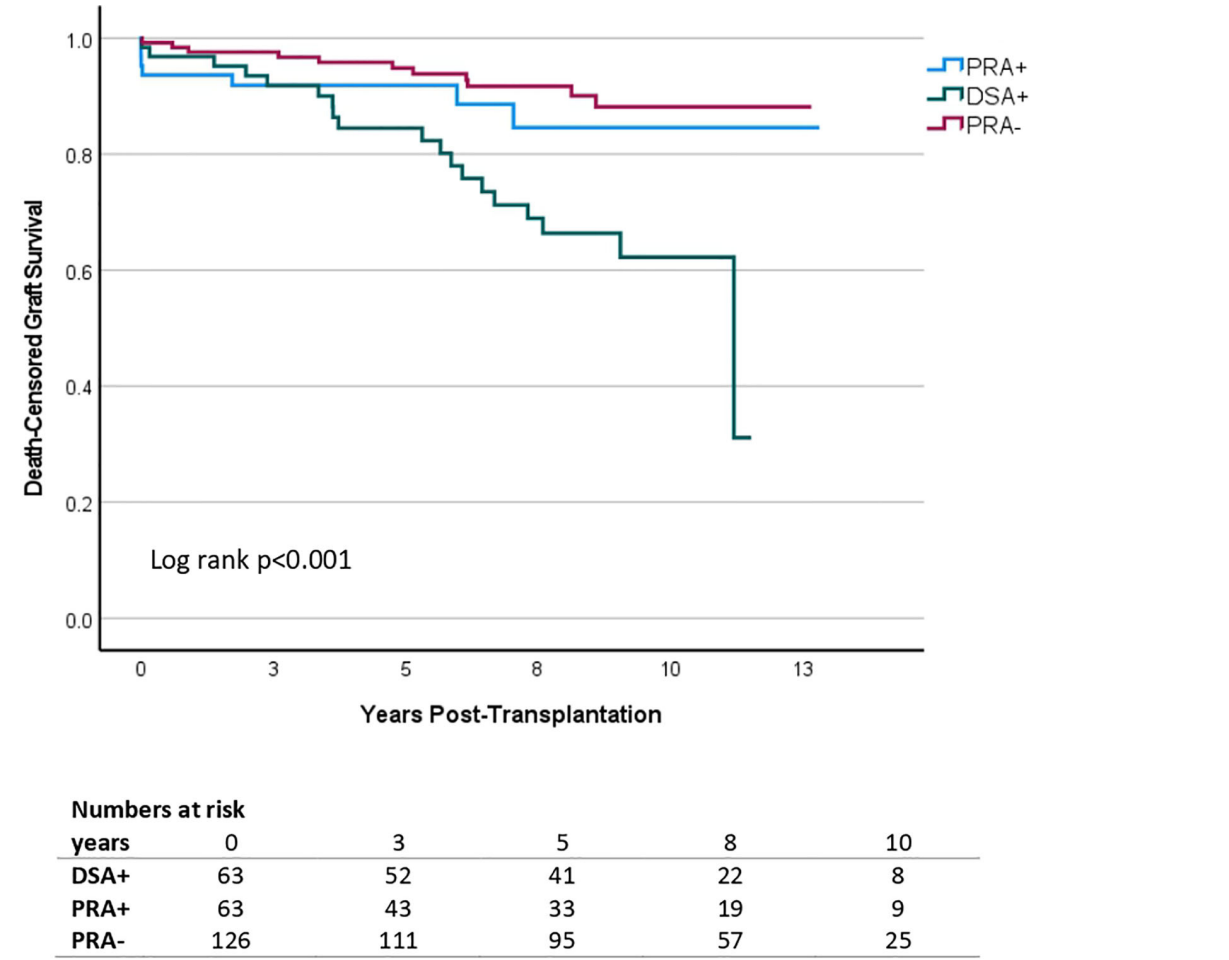
Kaplan Meier survival analysis of death-censored graft survival in living donor kidney transplantation according to the presence and type of pre-transplant anti-HLA antibodies. Patients with donor-specific antibodies (DSA+) were matched to equally immunized controls without DSA (PRA+) and non-immunized controls (PRA-). Additional matching variables were transplantation year, age of donor and recipient and donor-recipient relation. DSA+ (n=63) is depicted in green, PRA+ (n=63) in blue and PRA- (n=126) in red. The y-axis represents the proportion of individuals without graft failure, censored for death. The x-axis represents the time from transplantation in days.

There were 37 graft failures in the period observed, of which 19 in DSA+, 7 in pPRA+ and 11 in pPRA-. In Kaplan Meier survival analysis there was a significant difference in DCGS between DSA+, pPRA+ and pPRA- (**Figure 1**). In Cox regression analysis, DCGS was inferior in DSA+ compared to both pPRA+ and pPRA- recipients, with hazard ratio (HR) 2.38 [95% CI 1.00-5.70] for DSA+ versus pPRA+, and HR 3.91 [1.86-8.22] for DSA+ versus pPRA-. This corresponded to a 5-year DCGS of 82% for DSA+, 87% for pPRA+ and 94% for pPRA- (p=0.51 for DSA+ vs. pPRA+ and p=0.02 for DSA+ vs. pPRA-). After 10 years, DCGS was 31% for DSA+, 56% for pPRA+ and 69% for pPRA- (p=0.10 for DSA+ vs. pPRA+ and p=0.003 for DSA+ vs. pPRA-). In univariate analysis, DSA, peak PRA, mismatches on DR, retransplantation, recipient age, recipient BMI and induction therapy were associated with DCGS (**Table 2**). DSA remained to impact DCGS in multivariate analysis, with HR 4.54 [2.13-9.68]. The two sensitivity analyses either excluding recipients tested with OneLambda or excluding recipients tested with Lifecodes, did reveal the same negative impact of DSA on graft survival (**Supplemental Figure S2**).

**Table 2:** Univariate and multivariate Cox regression analysis for death censored graft survival in living donor kidney transplantation.

	HR	*p-value*
Univariate		
DSA	3.32 [1.74-6.33]	<0.001
Peak PRA (0-5%)		0.003
Peak PRA 6-84%	2.56 [1.20-5.48]	0.02
Peak PRA 85-100%	3.99 [1.72-9.25]	0.001
DR mismatch (0)	2.37 [0.84-6.69]	0.1
Retransplantation (none)	2.63 [1.38-5.02]	0.003
Recipient age	0.97 [0.94-0.99]	0.002
Recipient sex (female)	0.63 [0.33-1.91]	0.15
Recipient BMI	0.93 [0.85-1.01]	0.08
Multivariate		
DSA	4.54 [2.13-9.68]	<0.001
Age recipient	0.98 [0.96-1.00]	0.1

*Variables that were associated with DCGS with a p-value of p ≤ 0.1 in univariate Cox regression were included for multivariate analysis, as were age and sex of recipient. Backwards conditional method was the applied multivariate regression analysis method, as depicted in the lower part of the table.

HR: Hazard ratio; DSA: donor specific antibodies; PRA: panel reactive antibodies.

### Patient survival

Patient survival did not differ between DSA+, pPRA+ and pPRA- recipients in Cox regression analysis, with HR 0.93 [0.36-2.33] for DSA+ vs pPRA+ and 0.67 [0.30-1.49] for DSA+ vs pPRA- respectively. The corresponding 5-year patient survival was 85% for DSA+, 83% for pPRA+ and 94% for pPRA- respectively (p=0.75 vs pPRA+ and p=0.08 vs pPRA-).

### Antibody-mediated rejection

**Figure 2:**
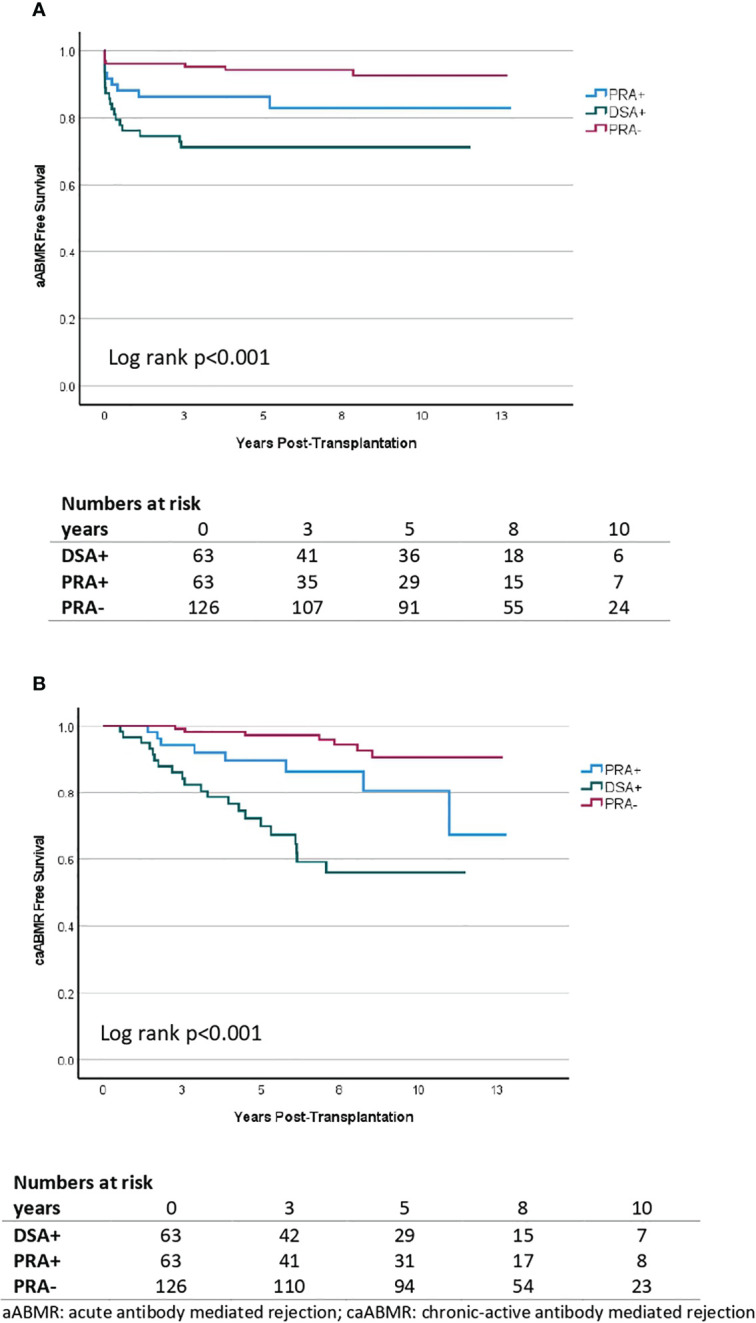
Kaplan Meier survival analysis of acute antibody mediated rejection **(A)** and chronic active antibody mediated rejection **(B)** in living donor kidney transplantation according to the presence and type of pretransplant anti-HLA antibodies. A comparison is made between donor-specific antibody (DSA) positive individuals (DSA+, n=63), highly immunized individuals (PRA+, n=63) and non-immunized individuals (PRA-, n=126). The y-axis represents the proportion of patients alive with a functioning graft without aABMR/caABMR. The x-axis represents the days post-transplantation.

There were 35 recipients who developed aABMR, of whom 18 in DSA+, 9 in pPRA+ and 8 in pPRA-. DSA+ recipients developed more acute ABMR (aABMR) in comparison to pPRA- recipients and at similar rates compared to pPRA+ recipients in Kaplan Meier survival analysis (**Figure 2A**). Univariate Cox regression analysis showed a HR of 1.92 [0.86-4.27] for DSA+ versus pPRA+ and HR 5.05 [2.20-11.62] for DSA+ versus pPRA-. Time to aABMR was not different across the three groups (p=0.19): the median days to aABMR was 61 [IQR 6-177] for DSA+, 28 [6-152] for pPRA+ and 9 [4-1162] for pPRA-. In all three groups, recipients that developed aABMR experienced more allograft loss (HR 3.84 [1.92-7.67] in Cox regression analysis. There were 36 recipients who developed caABMR, of whom 21 in DSA+, 8 in pPRA+ and 7 in pPRA-. Chronic active ABMR (caABMR) was also more common in DSA+ compared to pPRA+ and compared to pPRA- (Kaplan Meier survival analysis, **Figure 2B**). Univariate Cox regression analysis showed respectively HR 2.70 [1.20-6.13] and HR 8.44 [3.58-19.92]. Time to caABMR did not differ between DSA+ and pPRA+ (p=0.25), but was significantly shorter in DSA+ compared to pPRA- (p=0.04). Days to caABMR was 1134 for DSA+ [597-1826], 1238 for pPRA+ [650-2563] and 2501 for pPRA- [948-2940]. Also caABMR was associated with graft failure censored for death (HR 2.86 [1.46-5.63]) in Cox regression analysis.

### T-cell mediated rejection

**Figure 3:**
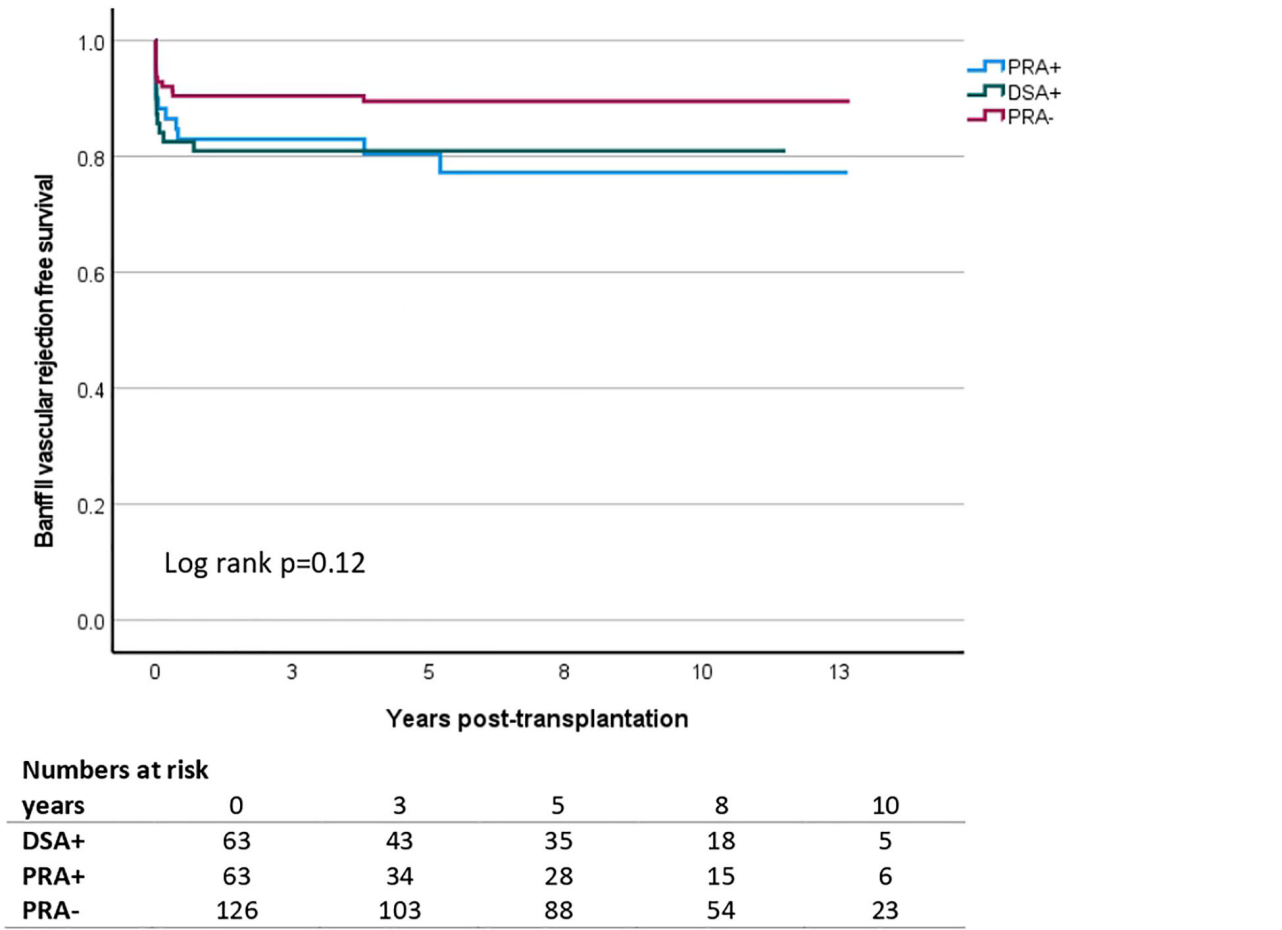
Kaplan Meier survival analysis of acute cellular Banff type II vascular rejection in living donor kidney transplantation according to the presence and type of pretransplant anti-HLA antibodies. A comparison made is across donor-specific antibody (DSA)-positive individuals (DSA+, n=63), highly immunized individuals (PRA+, n=63) and non-immunized individuals (PRA-, n=126). The y-axis shows the proportion of patients alive with a functioning graft without vascular rejection. The x-axis represents the days post-transplantation.

TCMR, at least Banff IA and IB cellular rejection, did not differ between the three groups. Banff type II vascular rejection occurred more frequently in both immunized groups, DSA+ and pPRA+ (Kaplan Meier survival analysis, **Figure 3**). HR was 1.99 [1.01-3.90] in Cox regression analysis. There was no additional effect of DSA on top of immunization with HR 0.93 [0.42-2.07] for DSA+ in comparison to pPRA+.

### Comparison within the DSA-positive group

#### DSA characteristics

Within DSA+ recipients, 41% had DSA against HLA class I, 38% against HLA class II and 21% against both HLA class I and II. 42 recipients (67%) had only one pretransplant DSA, 6 recipients (10%) had two DSA, four (6%) had three DSA and 11 (17%) had four or more DSA (**Table 3**). The median total MFI value was 6282 and the median MFI value of the immunodominant DSA was 4621. 56% of recipients had class I immunodominant DSA and 44% class II immunodominant DSA. In 53 out of the 63 recipients, flow-crossmatch results were available. Two subgroups were made: recipients with a positive flow-crossmatch (flow+) and recipients with a negative flow-crossmatch (flow-). Of all DSA+ recipients, 30% had DSA due to an RMM and 25% due to pregnancy. 18% were men with no RMM and 24% were females with no RMM and whose DSA could not be related to pregnancy (no partner or child HLA typing performed). One patient had both DSA from an RMM as well as from pregnancy. In one patient with DSA directed against HLA-DP, DSA origin could be not established as the previous donor was not typed for HLA-DP.

**Table 3:** Immunological characteristics of DSA+ patients.

	ABMR+	ABMR-	p-value
**Number of patients**	35	28	
Number of DSA			
1	22 [63%]	20 [71%]	0.47
2	3 [9%]	3 [11%]	0.77
3	2 [6%]	2 [7%]	0.82
4 or more	8 [23%]	3 [11%]	0.21
HLA Class			
Class I	11 [31%]	15 [54%]	0.08
Class II	15 [43%]	9 [32%]	0.38
Class I and II	9 [26%]	4 [14%]	0.27
Class immunodominant DSA			
Class I	18 [51%]	17 [61%]	0.46
Class II	17 [49%]	11 [39%]	0.46
MFI immunodominant DSA			
Median [IQR]	4016 [2504-9031]	5705 [3031-11069]	0.44
<5000	21 [60%]	12 [46%]	0.28
>5000	14 [40%]	14 [54%]	0.28
OL MFI >8000 or LC MFI >3000	12 [43%]	12 [34%]	0.49
Sum of MFI			
Median	6141 [2974-11456]	6469 [3481-12418]	0.58
<10.000	23 [68%]	17 [65%]	0.85
>10.000	11 [32%]	9 [35%]	0.85
SAB testing kit			
LifeCodes	15 [56%]	13 [56%]	0.94
OneLambda	12 [44%]	10 [44%]	0.94
Flow-crossmatch			
Flow +	11 [31%]	9 [32%]	0.54
Flow –	19 [54%]	13 [46%]	0.95
No flow result	5 [14%]	6 [21%]	0.46
Origin of DSA			
RMM	12 [34%]	7 [25%]	0.43
Pregnancy	10 [29%]	6 [21%]	0.52
Male, no RMM	5 [14%]	6 [21%]	0.46
RMM and pregnancy	0 [0%]	1 [4%]	–
Female, no RMM or HLA-typed pregnancy	7 [20%]	8 [29%]	0.43

DSA, donor-specific antibody; MFI, mean fluorescent intensity; flow, flowcytometric crossmatch; SAB, single antigen bead; RMM, repeated mismatch; OL, OneLambda; LC, LifeCodes.

### Allograft outcomes according to DSA characteristics

**Figure 4:**
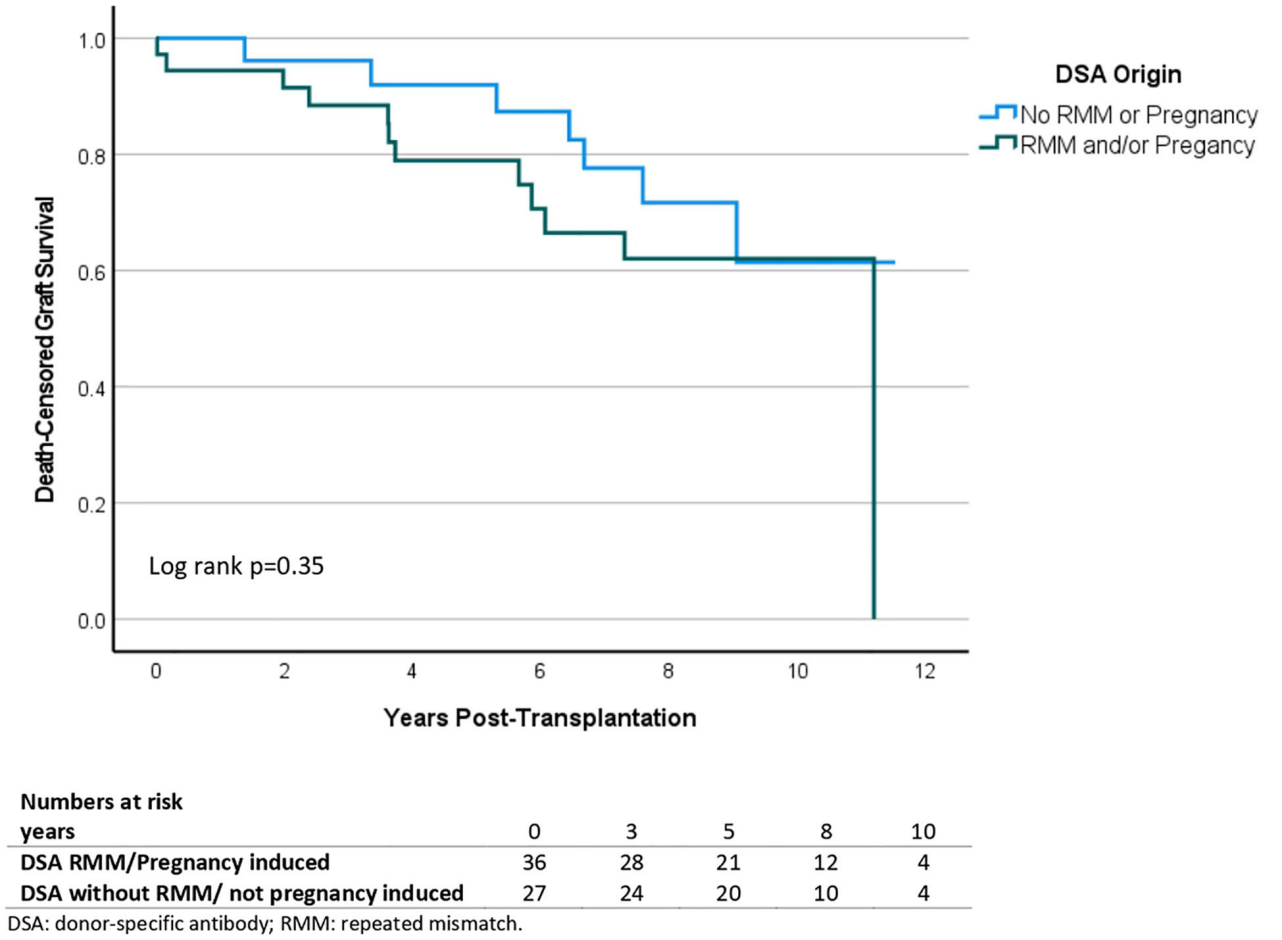
Kaplan Meier survival analysis of acute antibody mediated rejection according to origin of donor-specific antibodies. Comparison of DSA from a previous transplant (n=19), DSA from a pregnancy (n=16), DSA from both (n=1), men with DSA not from a previous transplant (n=11) and females with DSA not from a previous transplant or a pregnancy (n=15).

The risk of antibody mediated rejection (either acute or chronic-active) was not associated with DSA class, MFI values, flow-crossmatch results or origin of DSA (**Table 3**). Recipients with class I only DSA trended towards less ABMR (p=0.08). Another trend could be observed in the occurrence of early aABMR in recipients with DSA originating from pregnancy or a prior transplant (**Figure 4**). Of note is that four out of sixteen recipients with DSA from a pregnancy developed aABMR within 10 days after transplantation. There was no statistical difference in DCGS according to the origin of DSA in univariate Cox regression analysis (**Supplemental Table S1**).

Furthermore, a predefined sensitivity analysis was performed on DCGS in the DSA+ cohort. Firstly, when comparing flow+ and flow- recipients in Kaplan Meier survival analysis, DCGS was similar (p=0.83, **Supplemental Figure 1**). Also, when comparing DSA+flow- recipients with the immunized and non-immunized controls, DSA+flow- still had a negative impact on DCGS (**Supplemental Figure 1**). This was respectively with HR 2.76 [1.09-7.03] and HR 4.66 [2.05-10.56] in Cox regression analysis. Number of DSA, DSA class, the sum of the MFI values of DSA and the class of the immunodominant DSA (idDSA) did not impact DCGS (**Supplemental Table S1**).

There were 28 patients with MFI>5000. Paradoxically, MFI >5000 of the idDSA was associated with a significantly better DCGS than MFI <5000. However, as this is a retrospective analysis, two different Luminex assays were used: 56% of DSA+ recipients were tested in LifeCodes and 44% of DSA+ recipients were tested in OneLambda. In clinical practice, in our center we use the rule of thumb that MFI levels below 8000 in OneLambda generally correspond to MFI levels below 3000 in Lifecodes. Therefore we categorized the immunodominant DSA as either low (<8000 in OneLambda or <3000 in Lifecodes) or high (≥8000 in OneLambda or ≥3000 in Lifecodes). Results of this analysis still showed no association with death-censored graft survival according to MFI of the immunodominant DSA (Log rank p=0.58. **Supplemental Figure S3**).

## Discussion

In our cohort of (highly-) immunized living donor kidney transplant recipients, the presence of pretransplant DSA led to inferior death-censored graft survival, as compared to both matched immunized as well as non-immunized control recipients. More previous transplantations, higher peak PRA and more DR mismatches all correlated with this inferior graft survival and these characteristics were observed more frequently in DSA+ recipients. DSA remained of significant impact on DCGS in multivariate Cox regression analysis. DSA also led to more acute and chronic ABMR. Recipients with aABMR and caABMR had a worse DCGS. Banff ACR type II vascular rejection occurred more often in immunized individuals, but there was no additional effect of DSA on top of immunization status. There were no discriminating factors within DSA+ recipients indicating more safety of DSA.

The detrimental effect of DSA on graft survival as seen in our cohort, has also been described in several single- and multicenter studies (7–12). Our findings are in line with the observed inferior graft survival in a living donor transplantation cohort from Germany (9). Kamburova et al. (7) however did not find inferior graft survival in DSA+ living donor transplantations in the Dutch PROCARE consortium. This difference might be explained by different patient characteristics in our study. In the study by Kamburova et al., recipients with DSA had a mean peak PRA level of 44%, while for our study this was median 75%. Also, retransplantations were less common (48% versus 64% in our study).

Flow-crossmatch positivity on top of Luminex DSA positivity did not impact graft survival in our cohort, which is in contrast to other studies. In Orandi et al., the negative effect of DSA+ in comparison to DSA- controls was limited to flow+ and CDC+ recipients (11). In the same cohort, Motter et al. (17) did show more acute rejection in Luminex-only DSA+ recipients as compared to DSA- controls, but this had less impact on DCGS in comparison to acute rejection in HLA compatible transplantations. Of note is that in these studies all DSA+ recipients underwent desensitization. Desensitization practices vary, as the survival benefit in desensitized patients as compared to waitlisted patients seen in the United States was not substantiated in a large English registry (18, 19). Noble et al. describe in another European (French) desensitization cohort that graft survival was similar to evenly immunized patients who were transplanted with an HLA-compatible transplant, and a trend towards better patient survival (20). In our DSA+ cohort, 16% were CDC+ with desensitization and were excluded from the analysis.

Of note is that only 12% of DSA+ recipients received lymphocyte depleting induction. This differs from practices in for example the United States, where over 60% of recipients receive T-cell depleting induction therapy (21). Approaches to induction therapy vary substantially, and in many European countries basiliximab is first line therapy in the vast majority of recipients. Although KDIGO guidelines suggest using a lymphocyte depleting agent for kidney transplant recipients at high immunologic risk (22), this strategy was not adopted in the past in many (European) centers, as the evidence level of this recommendation was modest and most data were from deceased donor transplantation. The counter-intuitive finding that recipients with lower MFI values of the immunodominant DSA had worse DCGS might be explained by less DR mismatches compared to recipients with higher MFI levels. When trying to correct for the two different Luminex assays, this counter-intuitive finding was no longer present. Betjes et al. and Ziemann et al. also found no clear effect of MFI levels of DSA on DCGS and overall graft survival respectively (8, 9). However, Lefaucheur et al. found that in recipients with ABMR progressing to graft failure, higher MFI levels of DSA were present pretransplantation (10). MFI values in Lefaucheur et al. were however generally lower than in our study. Luminex based SAB methods were developed as qualitative analysis and can still only be considered semi-quantitative (6). There is no consensus on clinical relevance of MFI levels and viable cutoff values (23), and therefore SAB testing is accompanied by crossmatch testing. Class II DSA trended towards more ABMR in comparison to class I or combined class I and II DSA. This is an uncommon finding in literature, but results vary between studies. Kamburova et al. found worst outcomes for combined class I and II DSA, while Ziemann et al. and Lefaucheur et al. found no effect of DSA class (7, 9, 10). A Spanish study demonstrated a negative impact of class I DSA versus class II and versus combined class I and II (24). DSA from an RMM or pregnancy trended towards more and earlier aABMR. Literature has shown the deleterious effect of repeated mismatches on allograft outcomes in sensitized or DSA+ recipients, where DSA+ recipients with an RMM also had significantly more ABMR than DSA+ recipients without an RMM (25, 26). Lopes et al. demonstrated that previous transplantation and pregnancy gave stronger antibody formation in comparison to blood transfusions, expressed in MFI levels (27). In our study, however, MFI levels were not of influence. Apart from the limited number of DSA+ patients, only the pre-transplant DSA level was taken into account, not historical samples. MFI levels could well have been decreased over time since the immunizing event. Therefore pretransplant MFI levels might not reflect the capacity to mount a robust immune response to the allograft.

In DSA+ recipients, acute and chronic-active antibody mediated rejection in indication biopsies were more common than in immunized DSA- and non-immunized recipients. Vascular TCMR was more common in immunized recipients but there was no additional effect of DSA. Of further note is the low frequency of Banff I cellular rejection, comparable to non-immunized recipients. aABMR and caABMR were predictors of DCGS. This suggests that different pathological processes underlie these forms of rejection. This is in line with results from other studies (8, 10). The present data confirm pre-transplantation DSA and not height of PRA to be a risk factor for ABMR. Circulating antibodies do not seem to affect vascular rejection on top of immunization to HLA.

The strength of our study is the design with an immunized and a non-immunized control group. The chosen study design better enables unraveling of DSA impact on top of immunization status. The subgroup analysis in DSA+ allowed for an in depth analysis of DSA characteristics. The inclusion of only living donor kidney transplant recipients allowed for a valid comparison as prior research suggested differential effects of DSA in deceased versus living donor setting (7). A limitation is that assays to detect HLA antibodies have changed during the study period. Although the presence of DSA in the PRA+ group was ruled out by retesting Luminex SAB in this immunized group, the non-immunized control patients were not retested.

The relatively small sample size and retrospective nature is a limitation of our study. This is especially true for the subgroup analysis of the DSA+ recipients. Transplantation in the presence of pre-transplant DSA is, fortunately, a relatively rare scenario, only opted for in the absence of a compatible donor. A future study could therefore try to include a third control group of matched immunized candidates waitlisted for a deceased donor. Secondly, due to the retrospective design, bias by indication is a confounder. In addition, our study included ABO-incompatible transplantations, which may on itself have a negative effect on graft outcomes (28). This ABO-incompatibility, on the other hand, was equally distributed among the three groups. Lastly, DSA detection was performed with two different kits. Especially in the lower regions of MFI values, these might differ according to the kit used (29). To try to account for this limitation we performed two sensitivity analyses and an analysis with different cut-offs for both assays.

In conclusion, DSA led to inferior graft survival censored for death in our cohort of (highly-) immunized living donor kidney transplant recipients, compared to control recipients. No safe characteristics of DSA such as level of MFI could be deciphered. The presence of DSA per se reflects the capacity of the recipient to mount immunological damage to the allograft.

## Data availability statement

The raw data supporting the conclusions of this article will be made available by the authors, without undue reservation.

## Ethics statement

The studies involving human participants were reviewed and approved by Medical Ethical Committee Erasmus Medical Center Rotterdam. The patients/participants provided their written informed consent to participate in this study.

## Author contributions

AW, MR, MB and JW conceived the idea to perform this study. AW wrote the protocol for the study. TT, JG, JR, MK, JW, SB-S, DR and AW performed data collection for this study. TT, JG, JR, MB and AW performed statistical analysis of the acquired data. TT and AW wrote the manuscript. TT, DR, SB-S, JK, MK, MR, JR, JW, MB and AW revised and corrected the manuscript for the final version.
