# Supplementary material for: The detrimental effect of donor-specific antibodies is irrespective of its level in highly-immunized living donor kidney transplant recipients: A case-control series
Source: Front Immunol. 2023 Jan 10;13:1093359. doi: 10.3389/fimmu.2022.1093359 (PMC9873371; doi:10.3389/fimmu.2022.1093359)
Supplement: Supplementary file 1 [file DataSheet_1.docx]

| Supplemental Table S1: Univariate Cox regression analysis of death-censored graft survival according to pretransplant characteristics of donor-specific antibodies in living donor kidney transplantations. | | |
| --- | --- | --- |
|  | HR [95% CI] | P-value |
| N=63 |  |  |
| Number of DSA | 1.15 [0.89-1.48] | 0.29 |
| DSA class | 1.33 [0.75-2.37] | 0.33 |
| MFI immunodominant DSA (<5000) | 0.19 [0.06-0.66] | 0.01 |
| MFI total | 0.68 [0.24-1.92] | 0.47 |
| Immunodominant DSA class (class I) | 1.99 [0.80-4.96] | 0.14 |
| Origin of DSA  RMM or pregnancy vs. no RMM or pregnancy | 1.56 [0.61-3.96] | 0.35 |

DSA: donor-specific antibody MFI: mean fluorescent intensity; HR: hazard ratio; RMM: repeated mismatch

A
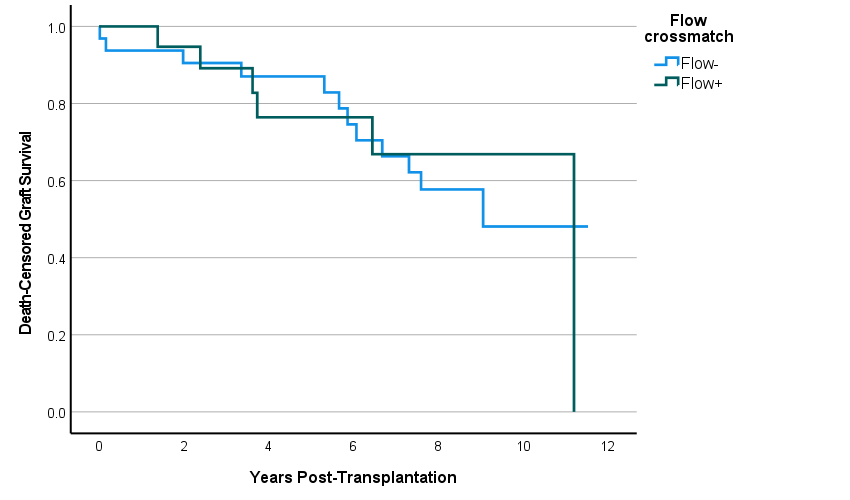


Log rank p=0.83

| Numbers at risk | | | | | |  |
| --- | --- | --- | --- | --- | --- | --- |
| years | 0 | 3 | 5 | 8 | 10 | |
| Flow-  Flow+ | 32  20 | 27  14 | 22  10 | 10  5 | 3  2 | |

B
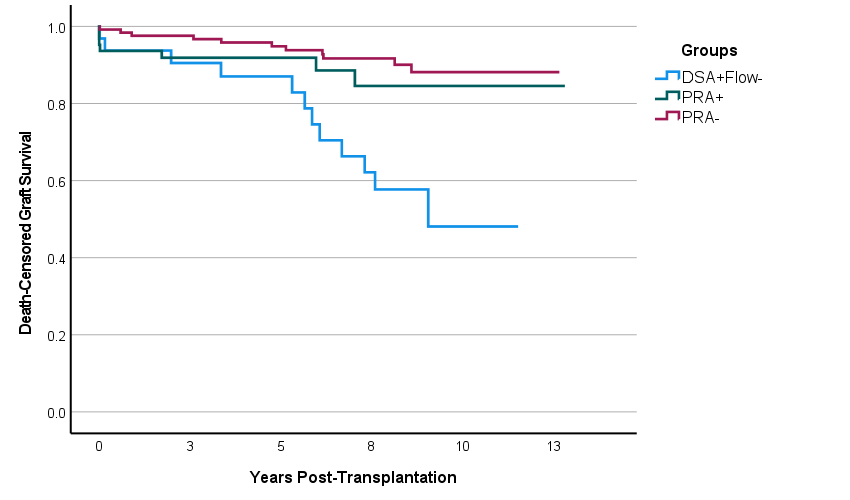


Log rank p<0.001

| Numbers at risk | | | | | |  |
| --- | --- | --- | --- | --- | --- | --- |
| years | 0 | 3 | 5 | 8 | 10 | |
| DSA+  PRA+  PRA- | 32  63  126 | 27  43  111 | 22  33  95 | 10  19  57 | 3  9  25 | |

**Supplemental Figure S1**: Kaplan Meier survival analysis of FACS+ DSA+ kidney transplant recipients versus FACS- DSA+ recipients. There is no difference in death-censored graft survival according to FACS-result. Figure B shows a sensitivity analysis of only FACS- DSA+ recipients, in comparison to pPRA+ and pPRA- recipients. Death-censored graft survival was significantly worse for FACS- DSA+ patients.

A
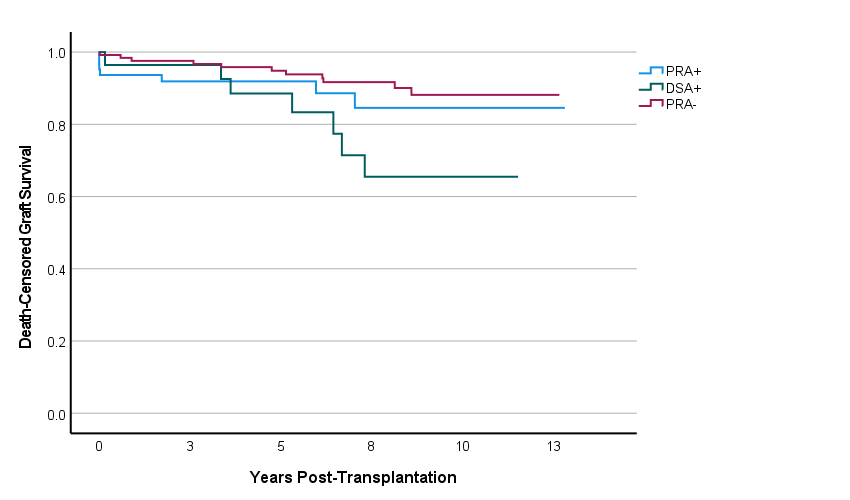


Log rank p=0.03

| Numbers at risk | | | | | |  |
| --- | --- | --- | --- | --- | --- | --- |
| years | 0 | 3 | 5 | 8 | 10 | |
| DSA+  PRA+  PRA- | 28  63  126 | 25  43  111 | 18  33  95 | 6  19  57 | 2  9  25 | |

B
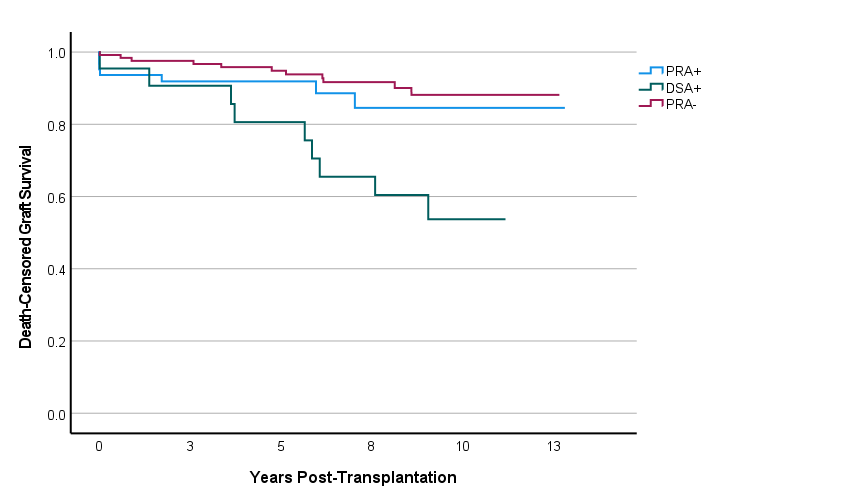


Log rank p<0.001

| Numbers at risk | | | | | |  |
| --- | --- | --- | --- | --- | --- | --- |
| years | 0 | 3 | 5 | 8 | 10 | |
| DSA+  PRA+  PRA- | 22  63  126 | 18  43  111 | 16  33  95 | 11  19  57 | 5  9  25 | |

**Supplemental Figure S2**: Kaplan Meier survival analysis of DSA+ kidney transplant recipients tested with two different Luminex assays, compared to pPRA+ and pPRA- recipients. Panel A demonstrates the comparison of DSA+ recipients tested with Onelambda (A) and Panel B demonstrates the comparison of DSA+ recipients tested with LifeCodes (B)., Death-censored graft survival was still significantly worse in both groups of DSA+ recipients (Log Rank p=0.03 for Onelambda and Log Rank p<0.001 for LifeCodes).


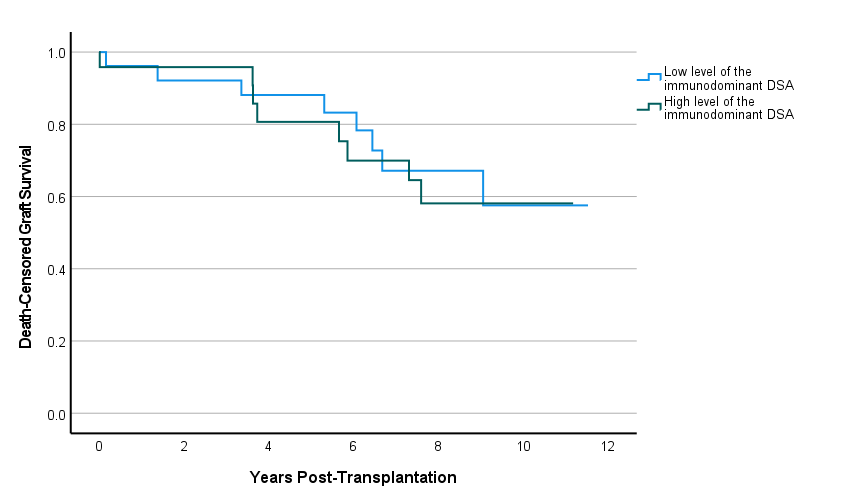


Log rank p=0.77

| Numbers at risk | | | | | |  |
| --- | --- | --- | --- | --- | --- | --- |
| years | 0 | 3 | 5 | 8 | 10 | |
| Negative MFI  Positive MFI | 26  24 | 23  20 | 19  15 | 10  7 | 4  3 | |

**Supplemental Figure S3**: Kaplan Meier survival analysis of the death-censored graft survival of DSA+ kidney transplant recipients according to a Luminex assay specific arbitrary cut-off for the immunodominant DSA:

For recipients tested with OneLambda the cut-off was MFI 8000 and for recipients tested with LifeCodes this was MFI 3000. There was no significant difference in death-censored graft survival between recipients with high level of immunodominant DSA (green) versus low level of immunodominant DSA (blue) p=0.77.
